# Buffalo Medical and Surgical Association

**Published:** 1890-09

**Authors:** W. H. Bergtold

**Affiliations:** Secretary


### BUFFALO MEDICAL AND SURGICAL ASSOCIATION.

Reported by W. H. BERGTOLD, M. D., Secretary.

THE regular monthly meeting was held in the parlors of the Iroquois hotel on Tuesday evening, August 5, 1890.

The president, Dr. A. A. HUBBELL, in the chair.

Dr. F. THOMA was elected to membership.

The following papers were read :

### HAY FEVER — ITS ETIOLOGY, PATHOLOGY, AND TREATMENT.

BY F. WHITEHILL HINKEL, M. D.,

Clinical Professor of Laryngology, Medical Department, University of Buffalo,

AND

FRANK HAMILTON POTTER, M. D.,

Lecturer on Diseases of the Nose and Throat, Medical Department, Niagara University.

### I.

### ON THE ETIOLOGY AND PATHOLOGY OF HAY FEVER.

By F. WHITEHILL HINKEL, M. D., Buffalo, N. Y.

IN THE past decade there has been much discussion of the essential character of hay fever and of its cause or causes. The classical investigations of Wyman in '72, Blackley in '73, and Beard in '76, established the observations that the disease is due to the pollen of various plants, and that only individuals of a neurotic temperament are affected. In '81 Daly advanced the statement that these cases present chronic diseased conditions of the nasal mucous membrane, the cure or relief of which relieved the attacks of hay fever. His observation of the relationship between diseased nasal conditions and hay fever was soon corroborated by scores of observers. A limited number affirmed the curative effect of intra-nasal treatment.

Two main pathological theories of undeniable and demonstrable conditions have sprung from these observations. The one regards hay fever as a pure neurosis, with exacerbations of a regularity similar to the periodicity that marks all nervous manifestations, physiological or pathological — as sleep, chills, etc. The other holds the manifestations of the hay fever paroxysm to be reflex from diseased intra-nasal conditions. The difference in methods of treatment to which this leads is obvious. Unfortunately these pathological theories, unlike the observations from which they spring, are not demonstrable. A post-mortem examination rarely includes in routine the nasal chambers, and hay fever victims live to suffer another day. I know of no record of a minute post-mortem on such a case.

Deductions from the results of treatment based on the one or the other hypothesis must be received with much allowance. We know that neurotics are enormously susceptible to suggestion, expectation, moral impressions, etc. Temporary improvement follows not infrequently in this class of cases from the most illogical and irrational procedures. Hence, because certain intra-nasal operations relieve certain cases at certain times, or for a certain time, it does not follow they will relieve all cases for all time. On the other hand, a change of residence to now well recognized localities, almost invariably gives relief to all cases. But, strange to say, all cases are not relieved by residence in the same locality. The influence is selective, so to speak, and each case is a law to itself outside of certain limits. This militates strongly against the pollen of any one plant being the sole exciting cause. And similar atmospheric conditions neither excite nor relieve all cases, as is well recognized in the kindred neurosis, asthma. A moderate view, which seems to be the wise one, in the elucidation of apparently discrepant phenomena reported by good observers, was formulated by Sir Andrew Clark in '85, and now generally accepted. It combines both hypotheses into a working theory, along the lines of which, in my opinion, the best results in treatment are to be obtained. According to this theory we have (1) a neurotic temperament, inherited or acquired; (2) a local condition of irritability or disease; and (3) an external exciting cause. This theory gains confidence from its comprehensiveness. It is applicable to any neurosis, or any reflex disturbance in any region.

The "golden mean" of opinion, then, holds that for the production of hay fever there must be present a neurotic constitution. The wider the investigator's range of observation, the firmer he holds this truth. The inferior races of men are not subject to this disease, or only with extreme rarity. It is confined largely, in the white race, to those whose nervous systems, environment and heredity have been "forced" in the hot-bed of modern life.

Next in importance in determining how the nervous explosions will manifest themselves to which this temperament is subject, is the local nasal disease. Often, it is a deflection of the septum; again, a hypertrophy of the nasal mucous membrane; occasionally a polyp; and frequently, only a general turgescence that between the attacks is not to be called disease. Areas of special irritability, not now necessary to particularize, at times seem to be the nasal *locus minimæ resistentiæ.* Any nasal abnormality causing pressure, in my experience, is the greal *local* factor in these cases. The just grounds upon which the reflex theorists urge the necessity of intra-nasal operations in many, if not a majority of cases, are obvious. If they allow the other factors due weight; if they worship not as a fetich the saw, trephine and cautery, good, or at most no evil, follows their method of treatment.

The third factor, the external irritant, is very interesting, and, unfortunately, well nigh universal. The emanations of flowers and various forms of vegetation are undoubtedly a great factor, but dust, sunshine and probably chemical and electrical states of the atmosphere play also their part. It is hopeless to try to escape them, except in limited regions and to a favored few. The susceptibility must be overcome by removing or weakening the influence of the other factors involved. In the treatment of the stay-at-home (of most interest to us), its consideration plays but a small though reasonable part.

It is the judicious consideration of the first and second factors (*i. e.*, the neurotic temperament and the nasal lesion) and their application to each case, studied according to the conditions it presents, that offer the best chances of an intelligent conception of these interesting yet exasperating cases, and that promise results satisfactory to physician and patient.

---

### II.

### ON THE TREATMENT OF HAY FEVER.

By FRANK HAMILTON POTTER, M. D., Buffalo, N. Y.

JUST as long as the pathology of hay fever is in the present chaotic condition its treatment will be uncertain and many times most unsatisfactory. I do not mean that there is no pathology of this disease ; on the contrary, there is a great deal, as the paper we have just listened to so clearly shows, but it has not yet reached that definiteness that can give us a fairly sure basis upon which to form a rational treatment. Some cases get well, — there is no doubt of that, — under careful and painstaking management, and such a result is one of great satisfaction to all concerned. Many cases, however, that have been reported as cured are really far from that condition, and could they appear here at this moment would demonstrate to you the uncertain meaning surrounding the word "cure" as it appears in our medical literature. All that we can be expected to do this evening is to show the line of treatment that should be adopted, in the light of our present knowledge, when we have to do with a case of hay fever, whether it comes to us under that plebian name, or under the more aristocratic one of rose cold, or, again, under perhaps the more scientific one of hyperesthetic rhinitis.

In the first place I desire to state that, as far as I can ascertain, no case of this disease has been cured by constitutional treatment alone. Many years elapsed between the year 1819, when the disease was first described by Bostock, and 1881, when Daly first called attention to the local lesions generally found in the nasal passages in this disease, and it is not too much to say that in all that time its treatment can be fairly described as *eminently unsuccessful.* On the other hand, local treatment alone is short-sighted and deceiving. Temporarily, its results are brilliant, but, as a rule, they do not last, and a case that has been cured in this way often returns to torment the operator and belie the statistics. It is just as bad to condemn local treatment as to condemn constitutional treatment, and the narrow generalist is as much bound down by the green withes of a confining theory, as is he who thinks the nose is the fixed point around which all things revolve.

Broadly, then, we must look at this disease from many points of view, and consider what we have learned :

I. As to the treatment during the attack.

II. As to the local treatment ; and

III. As to the general or constitutional treatment.

By the first, we seek to palliate the severity of the attack ; by the second and third, we seek to prevent the attacks recurring. It is now very generally conceded that medical treatment should not be undertaken during the attack. The latter may be lessened in its severity in many ways, but thorough treatment should be postponed to the period of immunity.

We all appreciate to-day that change of locality will, in a large number of cases, prevent or stop an attack. This is a measure that cannot be employed by many, either on account of the expense or other reasons. When nothing prevents this indulgence, the periodical return of the disease is a most admirable excuse for a journey, and if locations can be found where the sufferer is free from it he would be foolish not to take advantage of that fact. Something can be done, however, to relieve the patient that must stay at home. He must obey, with perhaps more strict observance, the laws of personal hygiene than is necessary for one not a victim of the disease. He may be in certain directions more sensitive than others. For instance, he may take cold easily, and when this is so he should strive to make himself less liable to this, by the proper employment of the cold bath, the judicious selection of clothing, etc. Or certain articles of diet may disturb him, and then, of course, they should be avoided. We cannot repeat in detail what is so well known to the profession, but simply mention the importance of hygiene in the conduct of a case of hay fever, because we believe it has much to do with the comfort of the patient. During the attack the nasal passages are generally inflamed and very sensitive, and a watery mucus is constantly flowing from them. They should be washed out frequently with some bland, unirritating solution, and then the surfaces covered with a coating of an oil to protect them from dust. When dust or pollen is especially offensive, a small piece of fine sponge inserted into each nostril will still further protect them. Cocaine has, on account of its peculiar properties, come into almost universal use by hay fever patients. There is danger in its use, and when given to a patient he should be told that too frequent spraying of the nose with it will very likely disturb the nervous system and produce restlessness, sleeplessness, and the like. It should be applied after the nose is washed clean, before the oil is used. A two or four per cent. solution is all that is required.

Internally a combination of atrophia and morphia is useful. They must be given in small doses frequently repeated. Sulphate of atrophia 1-200 gr. and murate of morphia 1-32 gr. is a good porportion. It has, occasionally, been my good fortune to stop or prevent a threatened attack by this treatment. Success with it, however, is not universal, and it cannot be considered a specific.

We have now to consider the management of the patient when free from the disease.

Looking first to the local treatment, we find that we must seek to relieve a general hyperesthetic state of the nasal mucous membrane, and also in the vast majority of cases to remove lesions of an obstructive character. The means employed for the correction of the latter often serve to relieve the former, and thus it would seem that they are frequently dependent the one upon the other. These obstructive lesions are of many kinds, and may be found either of the hard or soft tissues—frequently they are of both. They may be slight and projecting or so large that they prevent breathing through the nostrils. They may be hypertrophic or neoplastic in their character. Whatever may be their organization, they should be removed. This is especially so if they cause contact between the walls of the nostrils. The pressure this contact produces is not only a source of local irritation, but causes reflex symptoms, widespread and aggravating. It is not our purpose to describe the methods now adopted to get rid of these obstructions. That belongs to the realm of the specialist. It will suffice if we are able to impress upon each one here, that these things must be looked into and corrected in order to give hay fever patients the benefit of the best treatment. We are not likely to control the disease unless this is done.

Where the operative treatment does not relieve the highly sensitive condition of the nasal mucous membrane, or again, where the latter exists without obstructive lesions requiring operations, then the hypersensitiveness can be controlled by mild and superficial alteratıon of the nerve ends found in the membrane. This is an important part of the treatment, frequently, in my opinion, overlooked in the management of these cases. Now, as to the general or constitutional treatment, simultaneously with the correction of the local disease we must ascertain in a systematic manner the life history of the patient, particularly as to hereditary influences, temperament, etc. In the majority of cases we find this disease appearing in persons of the so-called neurotic habit. They may also show other departures from the normal that must be taken into account. We must consider their personal hygiene, the diet, exercise, bathing and so on. In a word, we must strive to place them in a condition of the greatest resistance. This is so easily said and so hard to accomplish, that we must frequently be content with an approach at what we aim rather than its full realization. In addition to all this, drugs have here an important place and will well repay the difficulty in finding the proper combination for any given case. Nerve tonics and alteratives have the first consideration.

Mackenzie, of Baltimore,[1] who is inclined to consider the disease as a pure neurosis, relies much upon the following formulæ:

I. ℞—Zinc phosphid. . . . . . . . . . . . . . . . . . . . . . . . . gr. 1-16.
Quin. sulph . . . . . . . . . . . . . . . . . . . . . . . . . . gr. ii.
Ext. nuc. vom. . . . . . . . . . . . . . . . . . . . . . . . . gr. ¼.
M. Ft. pil. no I.
S. To be taken before meals.

II. ℞—Liq. Arsenic, et hydrag. iodid., gtt. iii., ad v.
S. In a wineglassful of water, after meals.

These are really valuable and will indicate the kind of remedies to be employed. Besides those in the above combinations, we find recommended by various observers belladonna, hydrocyanic acid, valerian, assafetida, musk, lobelia amber, the bromides and iodides, chloral, opium and hyoscyamus. Iodine, in some cases, will be found of great value. A very pleasant way to prescribe it is in the form of hydriodic acid. The important point, as already suggested, as far as drugs are concerned, is to find the formula that will act with benefit upon a given case. This will often tax the patience and skill of the physician, but frequently after repeated trials, success will follow when least expected. One or two other things deserve mention. The cold douche to the spinal column has a decided therapeutic value in these cases; most authors from Gordon, who first, in 1829, mentioned it, to Bosworth, have regarded it with great importance. The latter author makes some pertinent suggestions as to its application. It is something more than a cold bath. The water must be dashed against the spine either by means

1. Trans. American Laryngological Association, 1886.—p. 167.

of the shower bath or else by an assistant. A simple method is to sit in the bath-tub and press a sponge full of cold water over the back. The intermittent application of cold in this way acts as a general tonic to the whole nervous system. The patient feels better at once, and after becoming accustomed to it, considers the day not rightly begun without it. If this paper aimed to be exhaustive, many other details in regard to treatment would have to be studied. What has been said, however, will indicate to a slight extent the manner of dealing with hay fever cases. It is a broad subject, still far from settled. And this being so, it is becoming in any one undertaking the treatment of these patients to recognize its complexity, and above all to remember that various factors enter into it, that it has a local as well as a general element, and that the best treatment will consist of special and constitutional therapeutics combined.

DISCUSSION.

Dr. DeLancey Rochester laid stress upon the importance of always making a local examination in cases of hay fever. The nose and throat ought to receive a thorough going over in every case. It is also a good point to remember that some cases of hay fever may be due to anemia, and to prevent this being overlooked we ought always to examine the blood if we are at all puzzled in finding the cause of an attack. Operations for the correction of any nasal error ought always to be undertaken and insisted upon. He has never had any bad results from the use of cocaine. For constitutional effects he has had the best results from the use of quinine, hyoscyamus, and opium.

Dr. J. W. Grosvenor mentioned several cases, and spoke of one where the species of plant, whose pollen caused the hay fever attacks, differed from year to year. He had had very good results from the use of chloral.

Dr. F. W. Bartlett had experimented with various powders, vapors, etc., and amongst others, ozone. He had used simple cream as an emolient several times, and found it to work well.

Dr. Willard has tried nearly everything recommended for hay fever, and had not found one applicable to all.

Dr. C. A. Ring asked if electricity, otherwise than as a cautery, had ever been used as a local application to the nasal mucosa.

Dr. Potter, in closing, said that Dr. Rochester's point concerning the relation of anemia to hay fever was a good one. He had never used electricity in the manner suggested by Dr. Ring.

Dr. Roswell Park made some remarks on a recent visit that he had made to Pasteur's Institute in Paris.

Under the head of *prevailing diseases*, typhoid fever and much of the usual infantile maladies were reported.

It was announced that Dr. W. S. Tremaine would speak on Hernia at the September meeting.

